# Antitumor activity of *Cuphea ignea* extract against benzo(a)pyrene-induced lung tumorigenesis in Swiss Albino mice

**DOI:** 10.1016/j.toxrep.2019.10.004

**Published:** 2019-10-11

**Authors:** Sherien K. Hassan, Amria M. Mousa, Nermin M. El-Sammad, Abeer H. Abdel-Halim, Wagdy K.B. Khalil, Elsayed A. Elsayed, Nayera Anwar, Michael W. Linscheid, Eman S. Moustafa, Amani N. Hashim, Mahmoud Nawwar

**Affiliations:** a Department of Biochemistry, National Research Centre, Dokki, Cairo, Egypt; b Department of Cell Biology, National Research Centre, Dokki, Cairo, Egypt; c Bioproducts Research Chair, Zoology Department, College of Science, King Saud University, Riyadh, Saudi Arabia; d Department of Chemistry of Natural and Microbial Products, National Research Centre, Dokki, Cairo, Egypt; e Department of Pathology, National Cancer Institute, Cairo University, Cairo, Egypt; f Laboratory of Applied Analytical and Environmental Chemistry, Humboldt-University, Berlin, Germany; g October University of Modern Sciences and Arts, 6th October City, Egypt; h Department of Phytochemistry and Plant Systematics, National Research Centre, Cairo, Egypt

**Keywords:** *Cuphea ignea*, Plant phenolics, Benzo(a)pyrene, Lung tumorigenesis

## Abstract

Lung cancer has one of the highest mortality rates among various types of cancer and is the most frequent cancer in the world. The incidence of lung cancer is increasing rapidly, in parallel with an increased incidence of smoking. Effective chemoprevention may be an alternative strategy to control the incidence of lung cancer. Thus, the objective of current work was to ascertain the possible preventive and therapeutic efficacies of *Cuphea ignea* extract in a mouse model of lung tumorigenesis and its cytotoxicity toward the A549 human lung cancer cell line. Lung tumorigenesis was induced by the oral administration of benzo(a)pyrene (50 mg/kg b.w.) twice per week to Swiss albino mice for 4 weeks. Benzo(a)pyrene-treated mice were orally administered *C. ignea* (300 mg/kg body weight, 5 days/week) for 2 weeks before or 9 weeks after the first benzo(a)pyrene dose, for a total of 21 weeks. At the end of the administration period, various parameters were measured in the serum and lung tissues. The results revealed that the oral administration of benzo(a)pyrene resulted in increases in relative lung weight, serum levels of tumor markers (ADA, AHH, and LDH), and the inflammatory marker NF-κB, and a decreased total antioxidant capacity compared with the control. In addition, decreased levels of enzymatic and non-enzymatic antioxidants, with a concomitant increase in lipid peroxidation, metalloproteinases (MMP-2 and MMP-12), and the angiogenic marker VEGF were detected in lung tissues. Moreover, benzo(a)pyrene administration induced the upregulation of PKCα, COX-2, and Bcl-2 expression, with the downregulation of BAX and caspase-3 expression. *C. ignea* treatment alleviated all alterations in these parameters, which was further confirmed by the histopathological analysis of lung tissues. The findings of the current work provide the first verification of the preventive and therapeutic potentials of *C. ignea* extract against benzo(a)pyrene-induced lung tumorigenesis in mice.

## Highlights

• Phenolic compounds from *Cuphea ignea extract* were identified.
• Chemopreventive and chemotherapeutic potentials of *C. ignea* extract were demonstrated in mice.
• Tumor, inflammatory, oxidative stress, apoptotic and angiogenic markers were investigated.
• Histopathological analysis showed improved lung tissues after treatment with *C. ignea* extract.

## 1 Introduction

Lung cancer is one of the most prevalent malignant tumors worldwide [1]. It is a major cause of cancer-associated death, accounting for 18.4% of all cancer deaths, equivalent to approximately 1.76 million deaths in men and women, which represent a major public health problem [2]. Tobacco smoking is the most important etiological risk factor for lung cancer development. The consumption of cigarettes is responsible for the high incidence of lung cancer; an approximately 10-fold increase in the risk of developing lung cancer is attributed to long-term smoking, compared with nonsmokers [3]. The major constituents of tobacco smoke, which has a prominent role in the induction of lung cancer induction, are polycyclic aromatic hydrocarbons (PAHs) [4]. Benzo(a)pyrene [B(a)P] is one of the most potent PAHs and the first carcinogen to be identified in cigarette smoke [5]. It is often used to induce lung tumors in mouse models, as these tumors have molecular and morphological resemblances to those observed in humans [6]. Previous studies have proven that the toxicity of B(a)P is due to its intermediate metabolites and the oxidative stress induced by reactive oxygen species (ROS), which play a vital role in B(a)P-induced lung carcinogenesis [7].

Cancer chemoprevention is a promising approach to reducing the incidence and mortality of lung cancer through the use of dietary, natural, or pharmaceutical products to inhibit cancer initiation to prevent DNA damage, arrest tumor progression in premalignant cells, and metastasis reduction without undesirable side effects [8]. In recent years, the search has shifted to novel nutritive or non-nutritive plant phytochemicals with antioxidant and anticancer activity for cancer treatment and prevention [9]. Flavonoids, a naturally occurring group of polyphenolic compounds, afford a major source of chemical diversity and have been investigated for their chemoprevention and chemotherapeutic properties in many cancers [10].

Among the herbal resources belonging to Lythraceae family, the new genus *Cuphea,* which consists of approximately 260 species of herbaceous perennials and small shrubs and is considered the largest of 32 genera of Lythraceae [11]. *C. ignea,* the cigar plant (sometimes called the firecracker plant) is a tropical small, multi-stemmed evergreen shrub that produces deep orange to bright red tubular blooms, which appear to “burst”, similar to fireworks. It has small, elliptical, bright green leaves [12]. In our earlier study, we were the first to determine the phytochemical constituents present in the aqueous ethanolic extract of the aerial parts of *C. ignea* (under publication). Recently, we isolated a novel coumarin with a rare structure from the extract and reported the cytotoxic activity of the coumarin and the extract itself in the H23 and H460 lung cancer cell lines [13].

To the best of our knowledge, there are no previous studies that report the phenolic profile of the aqueous ethanolic extract of the aerial parts of *C. ignea*. Moreover, no experimental evidence has yet been reported to investigate its antitumor effect *in vivo*. Therefore, our aim was to isolate and identify phenolic profile of this extract and to evaluate the chemopreventive and therapeutic efficiency of *C. ignea* extract against B(a)P-induced lung tumorigenesis in a mouse model and the cytotoxicity to the A549 human lung cancer cell line.

## 2 Materials and methods

### 2.1 Plant collection and extract preparation

Fresh samples of *C. ignea* aerial parts were collected from 30 km north of Cairo (30.183748, 31.147463; Nurseries Qanater Charities for Ornamentals, Al-Qanater Al-Khayreyah, Al-Qalyubia Governorate, Egypt). Plant identification was performed by Prof. Dr. Salwa Kawashty at the National Research Center (NRC). A voucher specimen was deposited at the herbarium of the NRC (voucher number: C 182). *C. ignea* aerial parts were dried in the shade, crushed, and extracted with 70% (v/v) aqueous EtOH under reflux. The obtained eluent was dried under vacuum at 55 °C–60 °C, dissolved in EtOH, and fractionated by column chromatography. The chromatographic process was initiated with H_2_O/MeOH (9:1, v/v), and gradually increased in gradients of 10%. The bioactivity of the collected fractions was traced and the active column fraction(s) were then investigated by HPLC-MS, TLC, PC, and electrophoresis as well. The detected phenolics were separated and purified. For structure elucidation of the isolates, intensive HR-ESI-MS, 1D and 2D NMR were applied. HR-ESI mass spectra were measured by using a Finnigan LTQ FT Ultra mass spectrometer (Thermo Fisher Scientific, Bremen, Germany) equipped with a Nanomate SI interface (Advion Biosystems, USA). An electrospray voltage of 1.7 kV (+/−) and a transfer capillary temperature of 200 °C were applied. High-resolution product ions were detected in the Fourier transform-ion cyclotron resonance cell of the mass spectrometer. NMR spectra were acquired in DMSO-*d*_6_ by using a Bruker 400-MHz NMR spectrometer (Bruker Corp., Germany). Standard pulse sequence and parameters were used to obtain one-dimensional ^1^H and ^13^C NMR spectra, and two-dimensional COSY, HSQC, and HMBC spectra, respectively. Chemical shifts (δ**)** were measured in ppm; ^1^H NMR chemical shifts were relative to tetramethylsilane (TMS), and ^13^C NMR chemical shifts to DMSO-*d*_6_ and were converted to the TMS scale by the addition of 29.8.

### 2.2 Isolation and identification of the phenolics detected by HPLC/ESI-MS

The dried aqueous EtOH extract (115 g) was loaded onto a Sephadex LH-20 (900 g) column (120 × 7.5 cm). Elution was then started using H_2_O, followed by isocratic elution in 10% steps from 10% to 100% MeOH. After removal of the solvents, 10 fractions (I – X) were obtained. Compound **1** (23 mg) was crystallized from a concentrated Fraction I (22 g, eluted with H_2_O). Compound **2** was identified in Fraction II (3.1 g, eluted with 10% aqueous MeOH) through comparative paper chromatography and UV spectral analysis (Shimadzu UV–vis-1601 spectrophotometer, Kyoto, Japan) of a sample isolated by preparative paper chromatography (Prep. PC), using 6% AcOH as solvent. Compound **3** (24 mg) was crystallized from a concentrated ethanolic solution of Fraction III (3.6 g, eluted with 30%). Compounds **4** (12 mg) and **5** were desorbed by H_2_O-MeOH (40%) as Fraction IV (2.7 g). Each was separated purely from a Sephadex LH-20 column. Further purification was performed by the application of Prep. PC, using BAW (n-butanol:acetic acid:H_2_O, 4:1:5, upper layer) as the solvent, thus yielding samples of pure **4** (32 mg) and **5** (49 mg). Compounds **6** (44 mg), **7** (53 mg), and **8** were obtained from Fraction VI (3.3 g, eluted with 60% MeOH–H_2_O) through Prep. PC using 6% AcOH. Compounds **9** (24 mg) and **10** (951 mg) were separated from Fraction VIII, (4.1 g eluted with 80% aqueous MeOH), by repeated Prep. PC (thrice) using BAW as the solvent. Compounds **11**, **12**, and **13** were isolated from Fraction X (4.7 g, eluted with MeOH) by silica gel column fractionation, using a 1:1 mixture of ethyl acetate:n-hexane mixture for elution, followed by recrystallization from ethanol, affording samples of quercetin (16 mg), myricetin (11 mg), and ellagic acid (9 mg). ESI-MS, ^1^H and ^13^C NMR spectra were then recorded, interpreted completely, and confirmed by HR-ESI-MS and 2D NMR spectroscopy.

### 2.3 *In vitro* experiments

#### 2.3.1 Preparation of working solutions of *C. ignea* extract for cell culture

Stock solutions of 10 mg/mL *C. ignea* extract were prepared in DMSO, and then diluted with growth medium to prepare a series of working solutions with a final concentration range of 0.0–1000.0 μg/mL. Finally, working solutions were filter sterilized (0.22 μm filter, Millipore, USA).

#### 2.3.2 Cell line and culture conditions

A549 human lung adenocarcinoma cells (Sigma-Aldrich, USA), were propagated in DMEM containing 10% FBS, 100 × 1% antibiotic/antimycotic solution, and 3.6 g/L NaHCO_3_. The cells were incubated in a CO_2_ incubator (ShelLab, USA) in an environment of 5% CO_2_, 37 °C, and 95% humidity [14,15]. At the onset of the experiment, the cells were detached by the incubation with trypsin, centrifuged, and counted in accordance with standard protocols [16].

#### 2.3.3 Cell viability assays

##### 2.3.3.1 MTT assay

The MTT assay was performed in accordance with the methods described by Elsayed et al. [17,18]. This assay monitors the formation of the formazan following the reduction of MTT by mitochondrial dehydrogenases in living cells. Briefly, after trypsinization, the cells were seeded in 96-well plates, incubated at 37 °C for 24 h, then medium was replaced with fresh medium containing different concentrations of the prepared extract. Cells were incubated for another 24 h, then MTT was added, and the cells were incubated for another 4 h. The supernatant was discarded and DMSO was added to dissolve the precipitated formazan crystals. The absorbance of formazan, was read at 550 nm using a microplate reader (Thermo Scientific, USA), and the percentage viability was calculated.

The IC_50_ values were calculated from the linear regression of the calibration curve.

##### 2.3.3.2 NRU assay

This assay is based on determination of the lysosomal absorption efficiency of living cells toward neutral red stain (3-amino-7-dimethylamino-2-methyl-phenazine hydrochloride) in accordance with Borenfreund and Puerner [19]. The assay was conducted as for the MTT assay, but after the second incubation period, the medium contained in the tested extracts was removed and cells were washed twice with PBS, and then incubated for 3 h in medium supplemented with NR reagent. The medium was washed off rapidly with CH_2_O:CaCl_2_ solution (0.5:1.0, %), and the cells were then incubated for 20 min at 37 °C in a mixture of CH_3_COOH:C_2_H_5_OH (1:50%) to dissolve the dye; finally, the absorbance at 540 nm was measured. Control sets were run under identical conditions without the test compound.

#### 2.3.4 Morphological examination

Morphological changes were examined by using an inverted contrast microscope (Nikon Eclipse T500, Japan) at 10× magnification.

### 2.4 *In vivo* experiments

#### 2.4.1 Animals

Healthy male Swiss albino mice (body weight: 20–25 g) were used throughout the study. The animals were obtained from the animal house of the NRC, Cairo, Egypt, and maintained in a controlled environmental condition on a 12-h light/dark cycle. The animals were fed a standard pellet diet and water *ad libitum*. All animal experimental procedures were conducted in line with the institutional guidelines of the Animal Care and Use Committee of National Research Centre, Cairo, Egypt, and with the Helsinki Declaration of 1975, and its revisions in 2000 and 2008. The experimental protocol was approved by the Research Ethical Committee of the NRS, Cairo, Egypt, Protocol number 16/164, prior to the beginning of the study in 2016.

#### 2.4.2 Experimental design

The experimental animals were randomly divided into five groups; each group contained 10 mice.

**Group I** (Control): Mice were orally administered corn oil by gavage twice per week for 4 weeks.

**Group II** (B(a)P): Mice were orally administered B(a)P (50 mg/kg body weight in corn oil) by gavage twice per week for 4 weeks, for tumor induction [20].

**Group III** (*C. ignea*): Mice were orally administered *C. ignea* extract (300 mg/kg body weight in distilled water) by gavage five times per week for 21 weeks, to assess the toxicity of the extract.

**Group IV** (*C. ignea* Pre-treatment): Mice were orally administered B(a)P by gavage on the same schedule as Group II, along with *C. ignea* extract on the same schedule as Group III. Pre-treatment with *C. ignea* extract was started 2 weeks prior to the first dose of B(a)P administration and continued for 21 weeks.

**Group V** (*C. ignea* Post-treatment): Mice were orally administered B(a)P by gavage on the same schedule as Group II along with *C. ignea* extract on the same schedule as Group III. Post-treatment with *C. ignea* extract was started 9 weeks after the first dose of B(a)P administration and continued until the end of the experiment.

The mice were weighed at the beginning of the experiment, every 2 weeks during the experimental period, and before sacrifice.

#### 2.4.3 Blood and tissue sampling

At the end of the experimental period, mice were fasted overnight, and anesthetized by ether; subsequently, blood samples were collected from the retro-orbital plexus and kept at room temperature for 25 min. The blood sample was centrifuged at 3000 rpm for 15 min to separate the serum, and then stored at -80 °C until assayed. All animals were sacrificed by cervical dislocation and the body weight was recorded. The lungs were immediately excised, washed in ice-cold saline solution, blotted to dry on filter paper, and weighed. Each lung was dissected into three portions. One portion was homogenized in 0.1 mol/L potassium phosphate buffer (pH 7.4) at a ratio of 1:10 (w/v) using Tissue master TM125 (Omni International, USA). After centrifugation at 3000 rpm for 10 min, the clear supernatant was stored at -80 °C for use in the biochemical analysis. The second portion of the lung tissue was snap frozen at −80 °C to be used for molecular analysis. The third portion of the lung was fixed in 10% formalin for histopathological analysis.

#### 2.4.4 Assessment of relative lung weight

The relative lung weight (RLW) was calculated from the following formula:

$$RLW=\frac{Recorded\;lung\;weight}{\text{F}\text{i}\text{n}\text{a}\text{l}\;\text{b}\text{o}\text{d}\text{y}\;\text{w}\text{e}\text{i}\text{g}\text{h}\text{t}\;}\times \;100$$

#### 2.4.5 Biochemical analysis

##### 2.4.5.1 Serum marker enzymes

Adenosine deaminase (ADA) and aryl hydrocarbon hydroxylase (AHH) were assayed by ELISA kits (Glory Science Co., Ltd, China). In addition, lactate dehydrogenase (LDH) was assayed colorimetrically by using a kit from Spectrum Diagnostics, Egypt. All kits were used in accordance with the manufacturer’s instructions.

##### 2.4.5.2 Antioxidant and oxidative stress markers

Serum total antioxidant capacity (TAC) and glutathione peroxidase (GSH-Px) in lung tissue were estimated by using kits from Biodiagnostic Research Reagents, Egypt in accordance with the manufacturer’s instructions.

Catalase (CAT) activity in the lung tissue was assayed by monitoring the dismutation of H_2_O_2_, as described by Aebi [21]. CAT enzyme activity was expressed in micromoles of H_2_O_2_ decomposed per minute per gram of tissue used.

Reduced glutathione (GSH) as well was estimated in accordance with the method of Beutler et al. [22] after sample deproteinization in an equal volume of metaphosphoric acid. GSH concentration was expressed in mg/g tissue used.

Malondialdehyde (MDA), the major indicator of lipid peroxidation in biological samples, was measured in the lung tissues by using a thiobarbituric acid reactive substance assay according to Lef’evre et al. [23]. The values were expressed in moles of MDA per gram of tissue used.

##### 2.4.5.3 Metalloproteinase (MMP) activity

MMP-12 was quantified in lung tissues by its catalytic effect on the N-succinyl-trialanyl-*p*-nitroanilide substrate as described by Zay et al. [24].

MMP-2 enzymatic activity was quantified in lung tissue by using the gelatin zymography [25]. Homogenized lung tissues were separated on 7.5% SDS-PAGES gel containing 0.2% gelatin. After electrophoresis, gels were washed in 2.5% Triton X-100, then incubated overnight at 37 °C in reaction buffer (50 mM Tris−HCl, pH 7.5; 5 mM CaCl_2_; 1 μM ZnCl_2_). Gelatinolytic activity was visualized by staining the gel with 0.5% Coomassie Brilliant blue R-250, and then destaining until clear bands appear against dark background. Gelatinase activity relative to control samples was determined by densitometry using a Bio-Rad Gel Doc™ XR + System (Bio-Rad, USA) and Image Lab™ 4.0 Software.

##### 2.4.5.4 Tumor angiogenic marker

Vascular endothelial growth factor (VEGF), the most potent and prominent stimulator of tumor angiogenesis, was determined in lung tissue by an ELISA kit (Glory Science Co., Ltd, China) in accordance with the manufacturer instructions.

##### 2.4.5.5 Serum inflammatory marker

Nuclear factor kappa (NF-κB) was assayed by using an ELISA kit (Glory Science Co., Ltd, China) in accordance with the manufacturer’s instructions.

#### 2.4.6 Gene expression analysis

##### 2.4.6.1 Isolation of total RNA

Total RNA was isolated from lung tissues of male mice by using TRIzol® Reagent (Invitrogen, Germany) in accordance with the manufacturer’s instructions. One unit of RQ1 RNAse-free DNAse (Invitrogen, Germany) was used to digest DNA residues from isolated RNA and re-suspended in DEPC-treated water. The aliquots were used immediately for reverse transcription (RT) or stored at -80 °C.

##### 2.4.6.2 RT reaction

Complete Poly(A)^+^ RNA isolated from lung tissues was reverse transcribed into cDNA in a total volume of 20 μL by using RevertAid™ First Strand cDNA Synthesis Kit (Fermentas, Germany). The RT reaction tubes were transferred to the thermocycler (Applied Biosystem, USA) and subjected to the following reaction program: 25 °C for 10 min, 42 °C for 1 h, and a final denaturation step of 99 °C for 5 min. The obtained cDNA was stored at -20 °C or used immediately for amplification through quantitative real time-polymerase chain reaction (qRT-PCR).

##### 2.4.6.3 qRT-PCR

A StepOne Real Time PCR System (Applied Biosystem, USA) was used to determine the lung cDNA copy number. PCR reactions were set up in a 25 μL reaction volume in accordance with the manufacturer’s instructions for SYBR® Premix Ex Taq™ (TaKaRa, Biotech. Co. Ltd). The reaction program contained three steps. The first step was 95.0 °C for 3 min, the second step was 40 cycles of (a) 95.0 °C for 15 s, (b) 55.0 °C for 30 s, and (c), 72.0 °C for 30 s, and the third step (conducted to obtain a melting curve) comprised 71 cycles, which started at 60.0 °C and then was increased by approximately 0.5 °C every 10 s up to 95.0 °C. The sequences of the specific primers for each gene are listed in Table 1. The quantitative values of qRT-PCR of PKCα, COX-2, BAX, Bcl-2, and caspase-3 genes were normalized to the expression of β-actin. The relative quantification of the target to the reference was determined by using the 2*^−^*^ΔΔCT^ method, as follows:

ΔC_T (test)_*=* C_T (target, test)_*−* C_T (reference, test)_

ΔCT (calibrator) *=* C_T (target, calibrator)_*−* C_T (reference, calibrator)_

ΔΔCT *=* ΔC_T (Test)_*−* ΔC_T (calibrator)_.

**Table 1:** Primer sequences used for qRT-PCR amplification.

Gene	Primer sequence (5'–3')	References
PKCα	Forward: TGA ACC CTC AGT GGA ATG AGT	[26]
	Reverse: GGC TGC TTC CTG TCT TCT GAA	
COX-2	Forward: CTG TAT CCC GCC CTG CTG GTG	
	Reverse: ACT TGC GTT GAT GGT GGC TGT CTT	
BAX	Forward: ACA AAG ATG GTC ACG GTC TGC C	
	Reverse: GGT TCA TCC AGG ATC GAG ACG G	
Bcl-2	Forward: CTC AGT CAT CCA CAG GGC GA	
	Reverse: AGA GGG GCT ACG AGT GGG AT	
Caspase-3	Forward: TGA GCA TTG ACA CAA TAC AC	[27]
	Reverse: AAG CCG AAA CTC TTC ATC	
β-actin	Forward: CAC GTG GGC CGC TCT AGG CAC CAA	[26]
	Reverse: CTC TTT GAT GTC ACG CAC GAT TTC	

The relative expression was therefore calculated by using the formula 2*^−^*^ΔΔCT^.

##### 2.4.6.4 DNA fragmentation analysis

Apoptotic DNA fragmentation was qualitatively analyzed by the detection of the laddering pattern of nuclear DNA, as described by Lu et al. [28]. Briefly, lung tissues were homogenized, washed in PBS, and lysed in DNA extraction buffer overnight at 37 °C. The lysate was then incubated with DNase free RNase for 2 h at 37 °C, extracted three times in an equal volume of phenol/chloroform (1:1, v/v), and re-extracted with chloroform by centrifugation at 15,000 rpm for 5 min at 4 °C. The extracted DNA was precipitated in two volumes of ice-cold 100% ethanol containing 10% 3 M sodium acetate at -20 °C for 1 h and centrifugation at 15,000 rpm for 15 min at 4 °C. After washing in 70% ethanol, the DNA pellet was air-dried and re-dissolved in Tris−HCl/EDTA. The DNA was then electrophoresed on a 1.5% agarose gel and stained with ethidium bromide in Tris-acetate/EDTA buffer. A 100 bp DNA ladder (Invitrogen, USA) was included as a molecular size marker and the DNA fragments were visualized by exposure to ultraviolet transillumination and photographed.

#### 2.4.7 Histopathological analysis

The formalin-fixed lung tissue samples were processed routinely and embedded in paraffin wax to form paraffin block sections of 5 μm in thickness. The paraffin blocks were stained with hematoxylin and eosin (H&E) and examined by using a light microscope [29].

#### 2.4.8 Statistical analysis

Data were subjected to one-way analysis of variance followed by LSD multiple range test using IBM SPSS statistics data editor software, version 19. All data are expressed as the mean ± standard error, and a p value of < 0.05 was considered to indicate statistical significance.

## 3 Results

### 3.1 Determination of phenolics in *C. ignea* extract by HPLC/ESI-MS

**Fig. 1:**
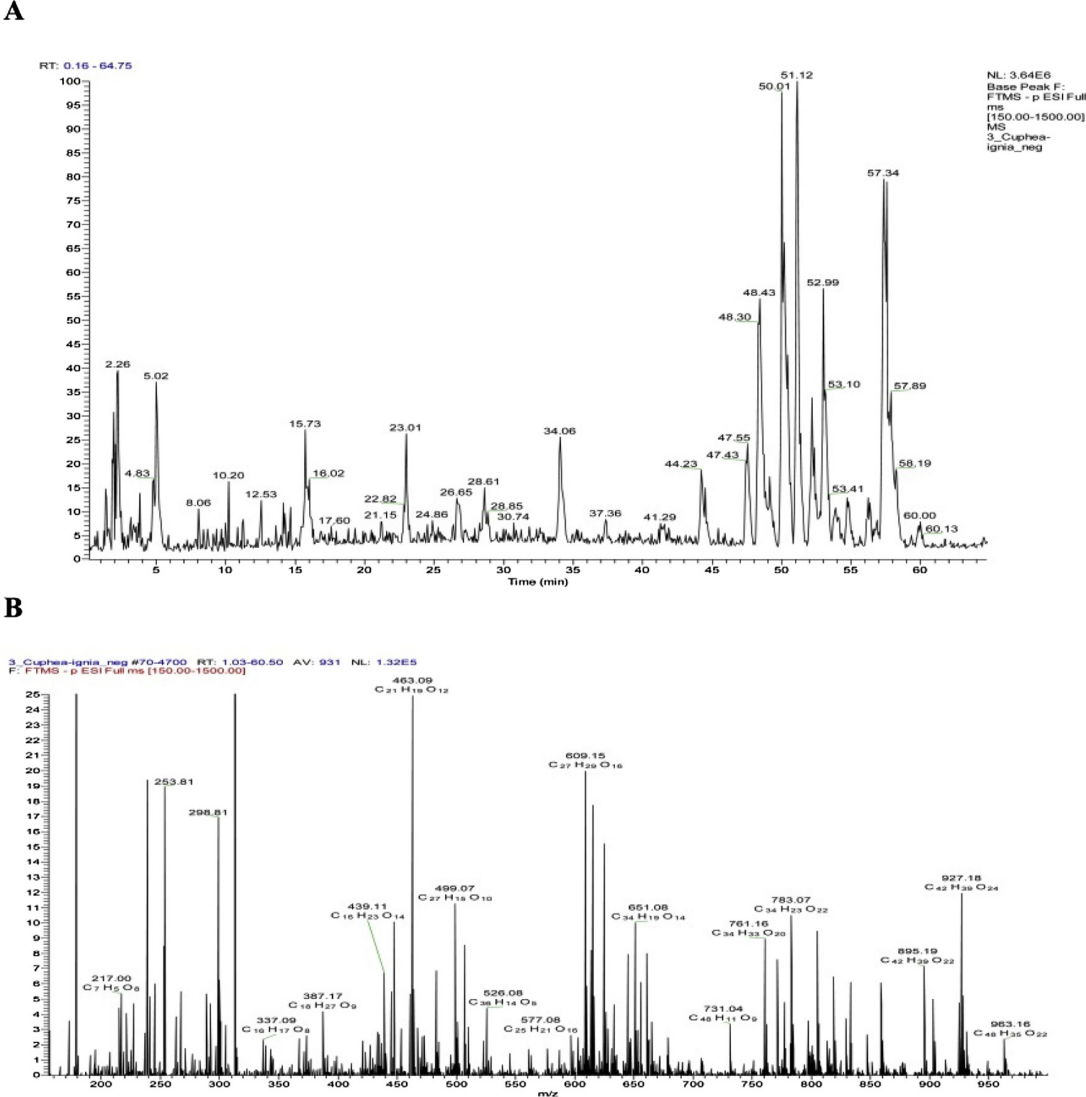
HPLC chromatogram of *C. ignea* extract (A), and the mass and molecular formula of the ions corresponding to its peaks (B).

**Fig. 2:**
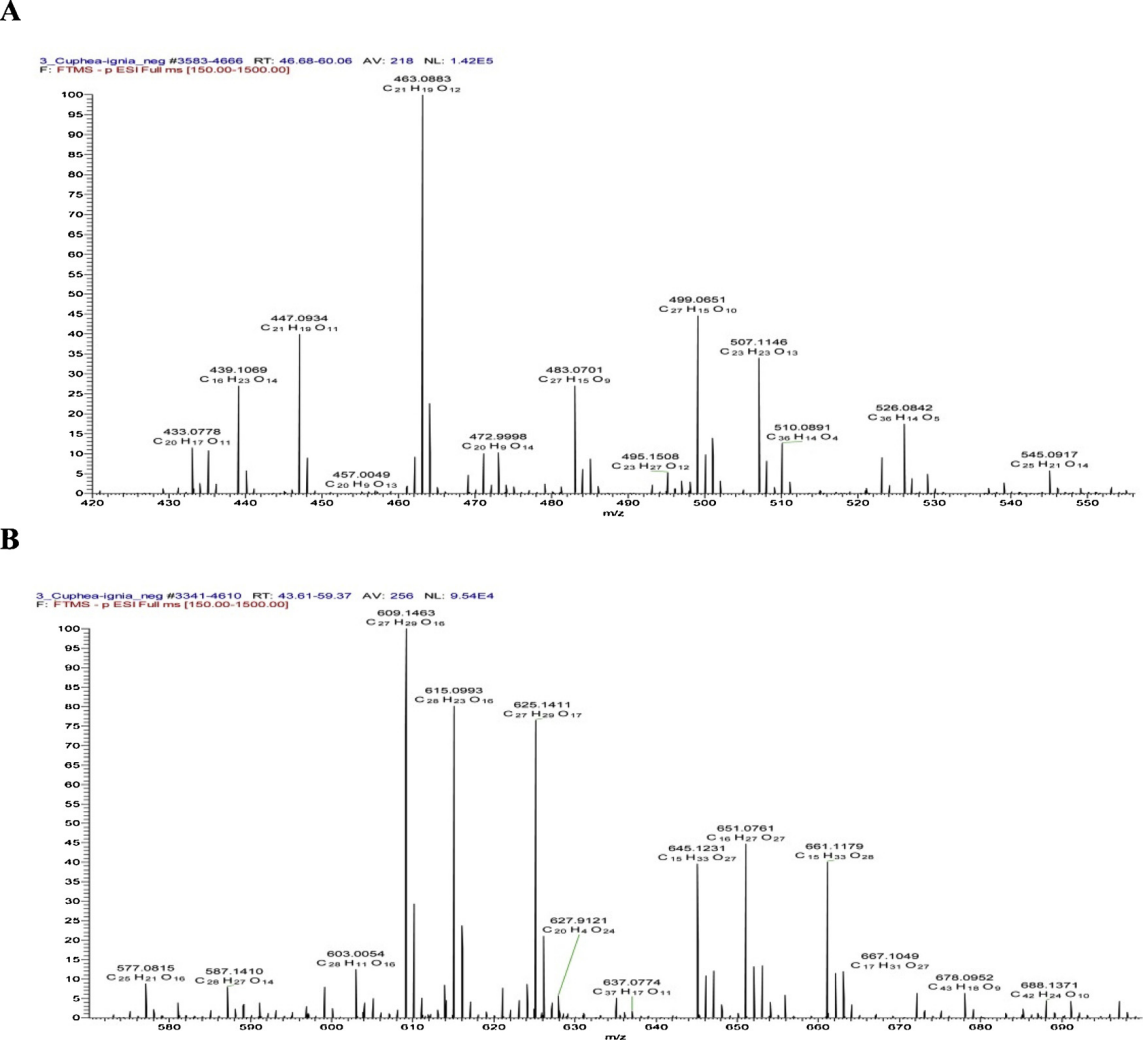
A–B). Selected ranges exhibiting negative molecular ions and a molecular formula in the HR-ESI-MS spectrum of *C. ignea* extract.

The phenolic compounds of the aqueous ethanolic extract of the aerial parts of *C. ignea* were found to be best fractionated by using a Sephadex LH 20 column eluted by MeOH/H_2_O mixture of decreasing polarities, a process which afforded 10 column fractions (I–X). Consequently, we were able to identify, isolate, and determine the structure of 13 metabolites (Table 2), three of which have been reported once before to occur in nature: namely, 7-hydroxy 3-methoxy coumarin 5-*O-*β-glucopyranoside (Cpd. 3) [13]; myricetin 7-methyl ether 3-*O*-xylosyl(1→2) rhamnoside (Cpd. 9); and myricetin 3′,5′-dimethyl ether 3-*O*-xylosyl(1→2) rhamnoside (Cpd. 10) [30]. These identified components were considered as targets. Their isolation, by means of consecutive polyamide S6, MCI gel, and repeated Sephadex LH-20 column fractionation, and Prep. PC, was applied. Ten polyamide S6 column fractions have been already obtained using a MeOH/H_2_O mixture with decreasing polarity as the solvent for elution. The isolation and purification of phenolics were monitored by two-dimensional paper chromatography. The ESI-MS, ^1^H, and ^13^C NMR spectra were then recorded, completely interpreted, and confirmed by HR-ESI-MS and 2D NMR spectroscopy. The HPLC chromatogram of *C. ignea* extract and HR-ESI-MS spectrum were shown in Fig. 1, Fig. 2.

**Table 2:** Identified compounds, molecular mass, and molecular formula.

Cpd.	Molecular ions (m/z)	Molecular formula	Identification
1	169.1207	C_7_H_6_O_5_	Gallic acid
2	183.7821	C_8_H_8_O_5_	Methyl gallate
3	369.3010	C_16_H_17_O_10_	7-hydroxy 3-methoxy coumarin 5-*O*-β-glucopyranoside
4	193.316	C_10_H_1o_O_4_	Ferulic acid
5	447.0935	C_21_H_19_O _11_	Quercetin rhamnoside
6	625.1410	C_27_H_29_O_17_	Quercetin sophoroside
7	463.0883	C_21_H_19_O_12_	Myricetin rhamnoside
8	761.1571	C_34_H_33_O_20_	Tellimagrandin I
9	609.1463	C_27_H_30_O_16_	Myricetin 7-methylether 3-*O*-xylosyl(1→2) rhamnoside
10	623.1690	C_28_H_32_O_16_	Myricetin 3’,5’-dimethyl ether 3-*O*-xylosy-(1 →2) rhamnoside
11	301.6401	C_15_H_10_O_7_	Quercetin
12	317.1601	C_15_H_10_O_8_	Myricetin
13	301.5105	C_14_H_6_O_8_	Ellagic acid

### 3.2 Effect of *C. ignea* extract on A549 cell viability

**Fig. 3:**
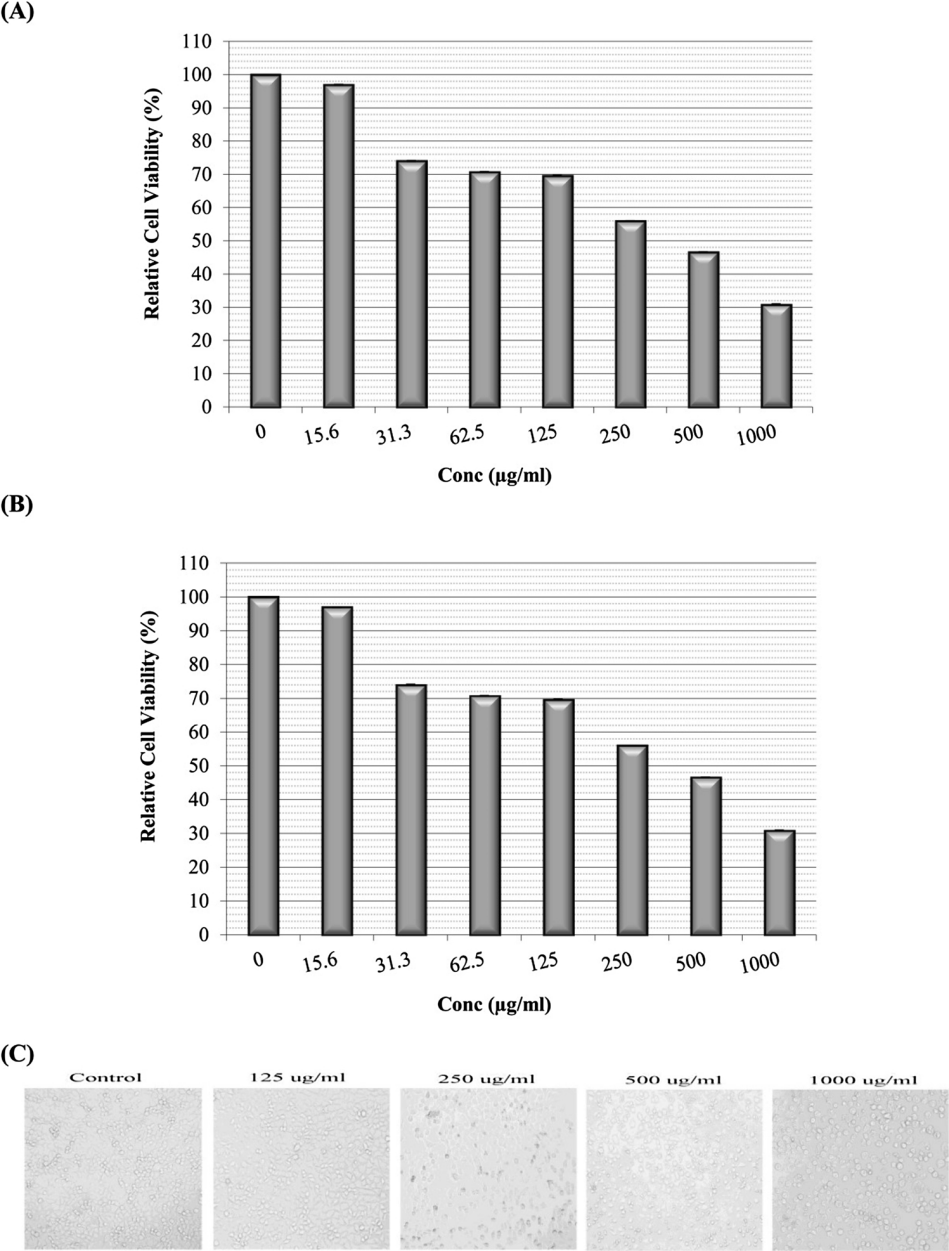
Cytotoxic effects of *C. ignea* against A549 cells. (A): MTT assay, (B): NRU assay, (C): morphological changes (Inverted contrast microscope, Nikon Eclipse T500, magnification 10×). Data are expressed as the mean ± SE of three replicates.

The cytotoxic effects of *C. ignea* extract were evaluated in A549 lung cancer cells by using MTT and NRU assays. The results obtained (Fig. 3) showed that *C. ignea* extract exerted a dose-dependent effect on cell viability: as the applied concentration increased, cell viability gradually decreased. The largest decrease in cell viability, *i.e.* the concentration at which toxicity was observed, occurred at the highest dose tested (1 mg/mL); in the MTT assay, cell viability was decreased by approximately 69.1% ± 2.9% at 1 mg/mL. The results of NRU assay were also comparable with the MTT results, with a decrease of approximately 80.2% in cell viability induced by 1 mg/mL extract. Thus, both assays confirmed the efficacy of *C. ignea* extract against the viability of A549 lung adenocarcinoma cells. The IC_50_ values of the *C. ignea* extract were 376.0 and 369.6 μg/mL in the MTT and NRU assays, respectively.

The morphological changes in A549 cells after treatment with different concentrations of *C. ignea* extract are shown in Fig. 3C. At concentrations of ≥125 μg/mL, the cell morphology started to change, and the changes also were proportional to the applied concentration, ranging from mild to severe changes. At lower concentrations, the cells began to shrink, started to detach, and then started to become free from the growth surface of the plates. As the extract concentration was increased, the cells started to round up, became flattened, and eventually died; conversely, the morphology of the control cells was unchanged.

### 3.3 Effect of *C. ignea* on body weight and RLW

**Fig. 4:**
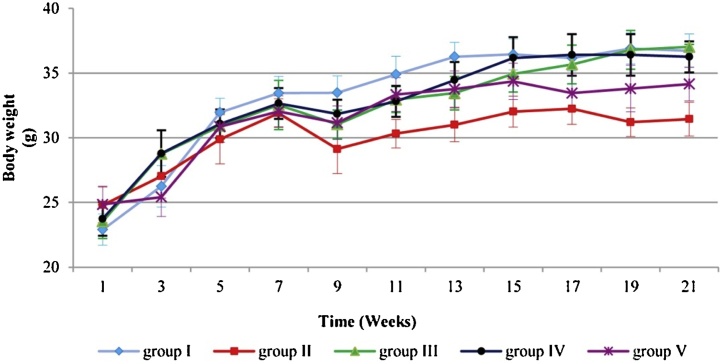
Effect of *C. ignea* on the mean body weight of control and treated mice. The values are expressed as the mean ± SE of six mice in each group.

The difference in mean body weight was not statistically significant between all treatment groups and the control. However, the final body weight in Group II was markedly lower (by 13.85%) compared with the control group; however, this reduction in the final body weight was considerably minimized by pre- and post-treatment with *C. ignea* (Fig. 4). The effect of *C. ignea* extract on the RLW of various groups is shown in Table 3. B(a)P treatment caused a significant increase in the RLW compared with control animals (Group I). Administration of *C. ignea* extract, in both the pre- and post-treatment groups, led to a remarkable reduction in RLW compared with the B(a)P group.

**Table 3:** Effect of *C. ignea* extract on relative lung weight of control and experimental mice.

Groups	Relative lung weight (%)	Relative lung weight increase (%)
Group I	0.59 ± 0.02	----
Group II	0.83 ± 0.05^a^	40.6%
Group III	0.63 ± 0.01^b^	6.77%
Group IV	0.64 ± 0.03^b^	8.47%
Group V	0.68 ± 0.01^b^	15.25%

Data are presented as the mean ± SE for six mice in each group. ^a^ P < 0.05 compared with the control group (Group I), ^b^ P < 0.05 compared with the B(a)P group (Group II).

### 3.4 Effect of *C. ignea* extract on the activity of tumor markers

The levels of tumor markers in the control and experimental animals are presented in Table 4. Our results revealed that the levels of ADA, AHH, and LDH in the B(a)P group were significantly increased, by 62.36%, 39.32%, and 62.16%, respectively, compared with the control group. In contrast, *C. ignea* pre-treatment significantly reduced the levels of ADA (35.26%), AHH (18.76%), and LDH (32.10%) compared with the B(a)P group. In addition, *C. ignea* post-treatment significantly reduced the levels of ADA (29.63%), AHH (13.04%), and LDH (30.24%) compared with the B(a)P group.

**Table 4:** The effect of *C. ignea* extract on the activity of tumor markers ADA, AHH, and LDH in the serum of control and treated mice.

Groups	ADA (pg/mL)	AHH (ng/mL)	LDH (U/L)
Group I	124.00 ± 4.41	3.13 ± 0.14	2337.8 ± 85.06
Group II	201.33 ± 24.31^a^	4.37 ± 0.34^a^	3791.2 ± 283.06^a^
Group III	119.49 ± 2.44^b^	3.19 ± 0.18^b^	2051.1 ± 159.23^b^
Group IV	130.33 ± 3.07 ^b^	3.55 ± 0.19^b^	2574.2 ± 89.41^b^
Group V	141.67 ± 13.05^b^	3.80 ± 0.08^a^	2644.4 ± 58.66^b^

Data are presented as the mean ± SE of six mice in each group. ^a^ P < 0.05 compared with the control group (Group I), ^b^ P < 0.05 compared with the B(a)P group (Group II).

**Fig. 5:**
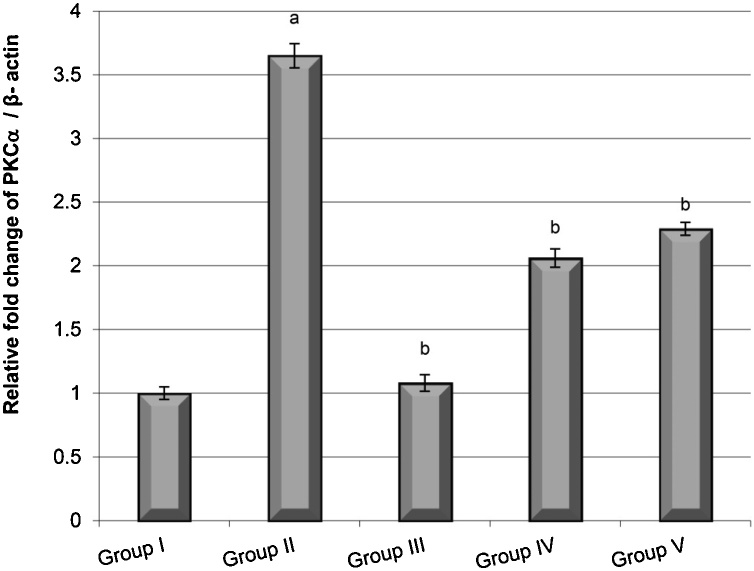
Gene expression of PKCα in the lung tissues of mice groups compared with that of β-actin determined by quantitative RT-PCR. Data are presented as the mean ± SE of six mice in each group. ^a^ P < 0.05 compared with the control group (Group I), ^b^ P < 0.05 compared with the B(a)P group (Group II).

The results also showed that the lung samples from animals in the B(a)P group exhibited significantly higher gene expression of PKCα than the control group. In contrast, the *C. ignea* pre- and post-treatment groups exhibited lower PKCα expression than the B(a)P group. The expression of PKCα in the *C. ignea* pre-treatment group was lower than that in the *C. ignea* post-treatment group (Fig. 5).

### 3.5 Effect of *C. ignea* extract on oxidative stress biomarkers

As shown in Table 5, MDA was significantly increased, by 98%, in the B(a)P group compared with the control group. The MDA levels in the *C. ignea* pre- and post-treatment groups was significantly reduced, by 34.84% and 20%, respectively, compared with the B(a)P group. In contrast, the B(a)P group showed significantly lower levels of TAC (59.00%), GSH (45.34%), GSH-Px (40.69%), and CAT (25.09%) compared with the control group. Pre-treatment with *C. ignea* extract significantly increased TAC, GSH, GSH-Px, and CAT, by 100%, 62.05%, 52.26%, and 20.79%, respectively, compared with B(a)P treatment. In the *C. ignea* pre-treatment group, TAC, GSH-Px, and CAT were restored to approximately the level in the control group. Post-treatment with *C. ignea* significantly improved TAC, GSH, and CAT by 82.92%, 39.51%, and 16.50%, respectively, compared to B(a)P treatment. However, *C. ignea* post-treatment caused a statistically insignificant increase in GSH-Px of 29.79%.

**Table 5:** The effect of *C. ignea* extract on serum level of TAC and levels of GSH-Px, CAT, GSH, and MDA in the lung tissues of control and experimental mice.

Groups	TAC (mM/L)	GSH -Px (U/g tissue)	CAT (U/mg tissue)	GSH (mg/g tissue)	MDA (nM/g tissue)
Group I	1.00 ± 0.05	53.49 ± 1.64	16.82 ± 0.57	225.27 ± 7.67	211.93 ± 16.15
Group II	0.41 ± 0.10^a^	31.72 ± 1.70^a^	12.60 ± 0.56^a^	123.13 ± 9.50 ^a^	419.72 ± 27.75^a^
Group III	0.99 ± 0.05^b^	53.17 ± 6.52^b^	17.34 ± 0.58^b^	237.97 ± 25.27 ^b^	255.57 ± 22.33^b^
Group IV	0.82 ± 0.14^b^	48.30 ± 3.38^b^	15.22 ± 0.80^b^	199.54 ± 6.70 ^b^	273.45 ± 17.63^b^
Group V	0.75 ± 0.07 ^b^	41.17 ± 3.21	14.68 ± 0.24^ab^	171.79 ± 13.87 ^ab^	336.48 ± 23.04^ab^

Data are presented as mean ± SE for six mice in each group. ^a^ P < 0.05 compared with the control group (Group I), ^b^ P < 0.05 for comparison to B(a)P group (Group II).

### 3.6 Effect of *C. ignea* extract on inflammatory markers

**Fig. 6:**
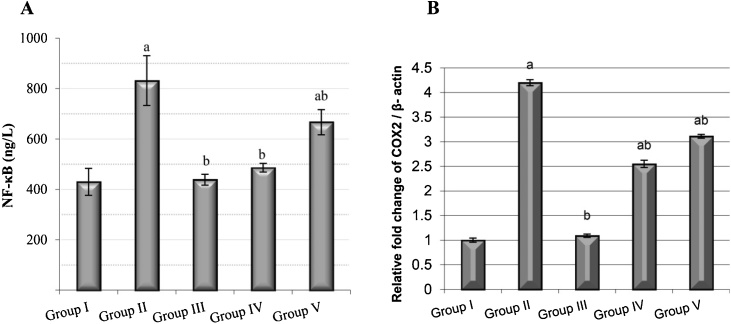
The anti-inflammatory effect of *C. ignea* extract on control and treated mice detected by (A): Serum NF-κB level and (B): COX-2 gene expression in the lung tissues determined by qRT-PCR. Data are presented as the mean ± SE for six mice in each group. ^a^ P < 0.05 compared with the control group (Group I), ^b^ P < 0.05 compared with the B(a)P group (Group II).

The NF-κB level was increased extensively, by 93.41%, in the B(a)P group compared with the control group. In contrast, pre-treatment and post-treatment with *C. ignea* extract reversed the increase, by 42% and 20%, respectively, compared with B(a)P group (Fig. 6A). A significant increase in the expression of the COX-2 gene was observed in the B(a)P group compared with the control group. *C. ignea* supplementation caused a significant decrease in the expression of COX-2 in *C. ignea* pre- and post-treatment groups compared with the B(a)P group (Fig. 6B).

### 3.7 Effect of *C. ignea* extract on MMP activity

**Fig. 7:**
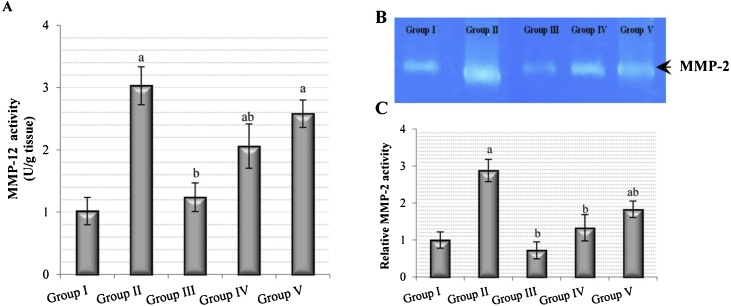
The effect of *C. ignea* extract on lung matrix metalloproteinases (MMPs) in control and treated mice animals. (A): MMP-12 activity in lung tissues. (B): Gelatin polyacrylamide gel showing MMP-2 activity in lung tissues. (C): Densitometric analysis of MMP-2 activity in zymogram obtained by using an image analysis program; the levels are expressed in arbitrary units relative to the MMP-2 value in the control group. Data are presented as mean ± SE for six mice in each group. ^a^ P < 0.05 for comparison to control group (Group I), ^b^ P < 0.05 for comparison to B(a)P group (Group II).

MMP-12 activity in the B(a)P group was significantly increased, by two-fold, compared with the control group. The *C. ignea* pre-treatment group showed significantly lower activity, by 32%, and the *C. ignea* post-treatment group caused an insignificant decrease in MMP-12 activity, by 15%, compared with the B(a)P group (Fig. 7A). Gelatin zymography detected MMP-2 as bright lysis zones (Fig. 7B). MMP-2 activity was significantly higher, by two-fold, in the B(a)P group compared with the control group; in addition, the *C. ignea* pre- and post-treatment showed a significant decrease in MMP-2 activity, by 53.81% and 36.45%, respectively, compared with the B(a)P group (Fig. 7C).

### 3.8 Effect of *C. ignea* extract on angiogenic marker

**Fig. 8:**
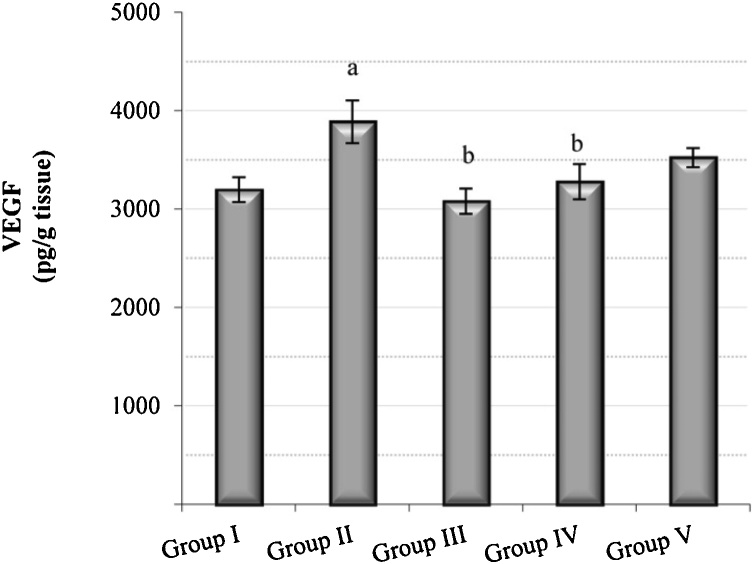
The effect of *C. ignea* extract on VEGF in the lung tissues of control and treated mice. Data are presented as the mean ± SE of six mice in each group. ^a^ P < 0.05 compared with the control group (Group I), ^b^ P < 0.05 compared with the B(a)P group (Group II).

A significant increase of 21.6% in the VEGF level was observed in the B(a)P group compared with the control group, whereas the *C. ignea* pre- and post-treatment groups showed lower VEGF levels, by 16% and 10%, respectively, compared with the B(a)P group (Fig. 8).

### 3.9 Effect of *C. ignea* extract on the expression of apoptotic markers and apoptotic DNA fragmentation

**Fig. 9:**
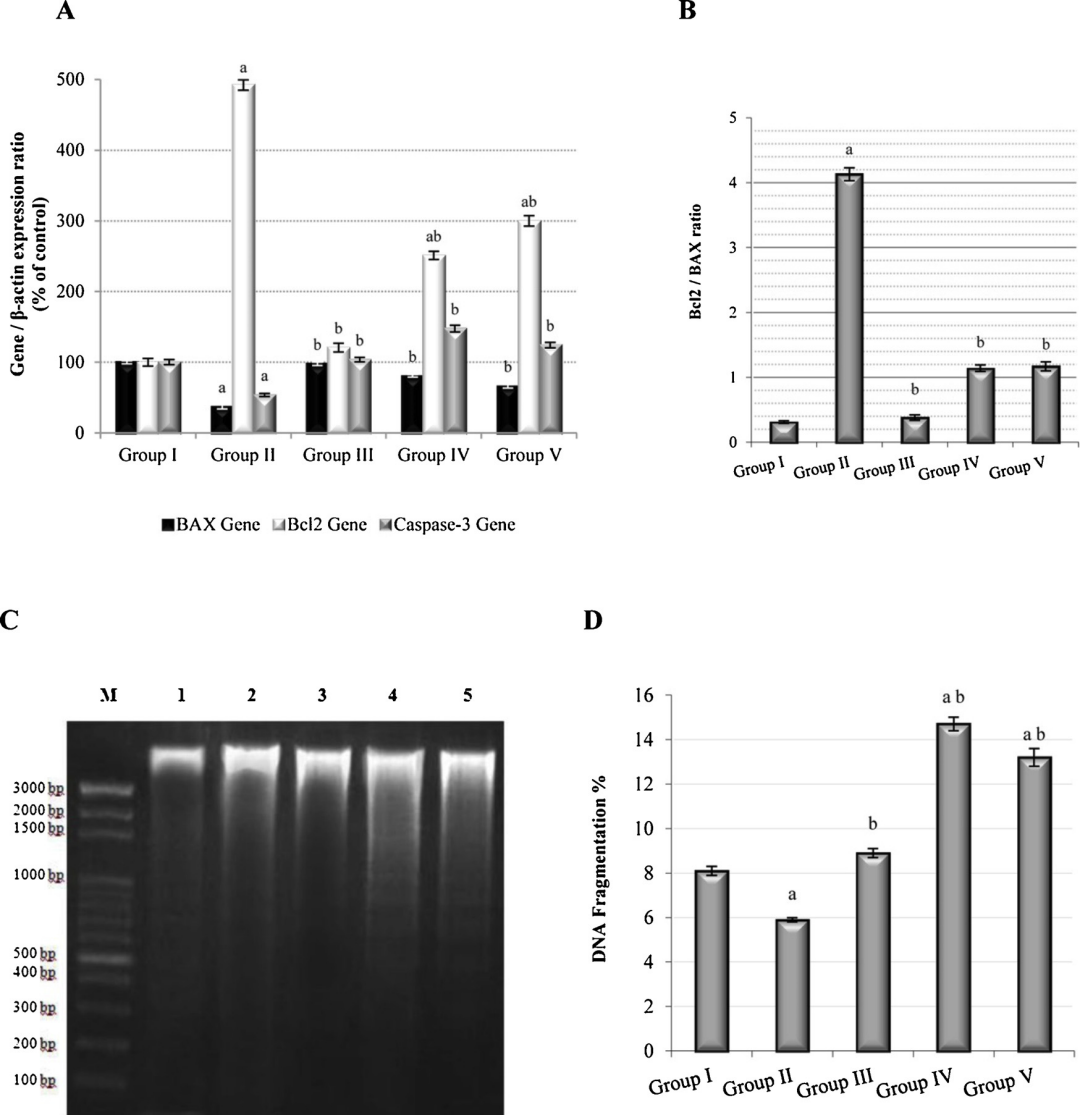
Effect of *C. ignea* extract on apoptotic processes in the control and experimental mice. A): Gene expression of BAX, Bcl-2, and caspase-3 in the lung tissues relative to that of β-actin, as determined by qRT-PCR. B): Bcl-2/BAX ratio. C): Densitometric analysis for DNA fragmentation in the lung tissue using agarose gel electrophoresis. M: DNA ladder, Lane 1: Control samples, Lane 2: B(a)P-treated samples, Lane 3: *C. ignea*-treated samples, Lane 4: *C. ignea* pre-treated samples; and Lane 5: *C. ignea* post-treated samples. D): Percentage of DNA fragmentation in the lung tissue. Data are presented as the mean ± SE of six mice in each group. ^a^ P < 0.05 compared with the control group (Group I), ^b^ P < 0.05 compared with B(a)P group (Group II).

The results revealed a significant decrease in the expression of pro-apoptotic genes (BAX and caspase-3) and a significant increase in the expression of an anti-apoptotic gene (Bcl-2) in the B(a)P group compared with the control group. In contrast, pre and post-treatment with *C. ignea* extract resulted in a significant increase in BAX and caspase-3 gene expression and a significant decrease in Bcl-2 gene expression compared with B(a)P treatment (Fig. 9A). A significant increase in the Bcl-2/BAX ratio was observed in the B(a)P group compared with the control group. Pre- and post-treatment with *C. ignea* resulted in a significant reduction in the Bcl-2/BAX ratio compared to the B(a)P treatment (Fig. 9B). Moreover, DNA fragmentation was significantly decreased in the B(a)P compared with the control group. In contrast, DNA fragmentation was significantly higher in the *C. ignea* pre- and post-treatment groups than in the B(a)P group.

### 3.10 Effect of *C. ignea* extract on lung histopathology

**Fig. 10:**
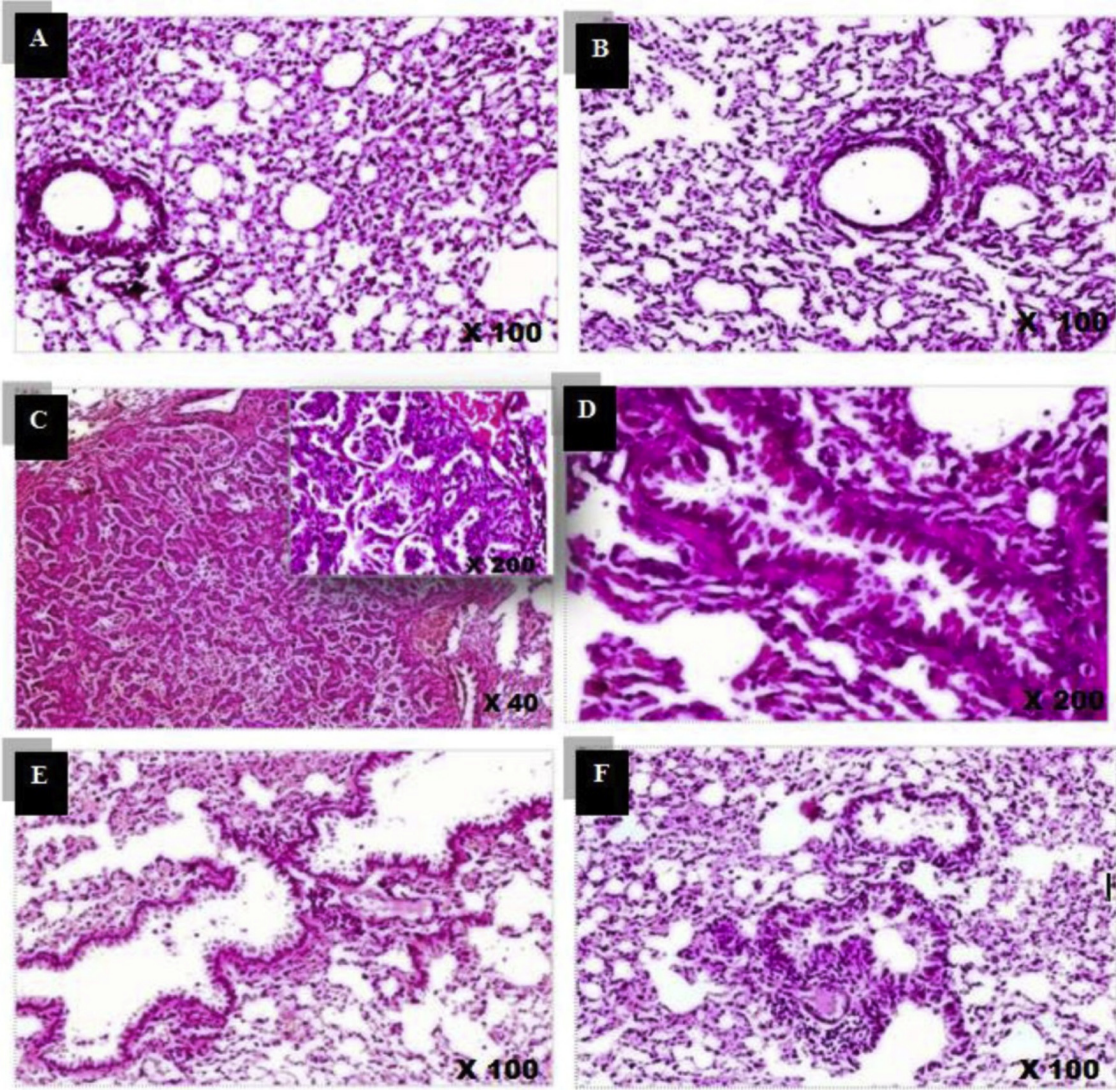
A representative sample of the histopathological findings in the lung sections of control and treated mice (H&E staining). A): Control section showing intact normal histological features. B) *C. ignea* lung section showing preserved cytological architecture. C), D): B(a)P group exhibited a well-defined tumor formed of irregular papillae lined by hyperplastic epithelial cells. E): Mice pre-treated with *C. ignea* showed mild multifocal interstitial inflammatory exudate. F): Mice post-treated with *C. ignea* exhibited bronchioles with evident hyperplastic epithelial lining, as well as moderate diffuse intervening exudate of inflammatory cells.

The histological analysis clearly indicated normal histological features with normal patent alveoli. The intervening bronchioles were lined with an intact normal pseudo-stratified columnar ciliated epithelium in the control and the *C. ignea-*treated sections in Groups I and III. B(a)P-treated mice (Group II) exhibited a well-defined tumor, with a surrounding intact fibrous capsule. The tumor was formed of compact back-to-back papillae, lined by stratified columnar epithelial cells. Some areas showed diffuse marked epithelial cells infolding with minimal dysplasia. The mice pre-treated with *C. ignea* extract showed normal epithelial lining of the bronchioles. The alveoli exhibited normal cytological patterns and normal cell alignment. The interstitium displayed mild multifocal exudate of inflammatory cells, with mainly mature lymphocytes. The *C. ignea* post-treatment group showed bronchial hyperplasia with some epithelial cells infolding, as well as the exudate of inflammatory cells (Fig. 10).

## 4 Discussion

The utilization of natural products for the management of cancer is one of the currently established strategies [31,32]. Plant-derived phenolic compounds have demonstrated the importance of their role in cancer prevention and treatment, which occurs mainly due to their ability to control the levels of ROS, induce differentiation and apoptosis, block signaling pathways, and suppress cell proliferation and angiogenesis [33,34]. As a consequence, phenolic compounds inhibit the initiation and progression of various cancer cells [35,36]. In this study, we have presented the first investigation of the phenolic profile of the aqueous ethanolic extract of the aerial parts of *C. ignea*. The current investigation aimed to elucidate the protective role of aqueous ethanolic extract of *C. ignea* in B(a)P-induced lung tumorigenesis in mouse model.

Results obtained for the *in vitro* cytotoxic activities showed that *C. ignea* extract has potential cytotoxic activity against human lung cancer cell line (A549). The cytotoxic activity may be due to the presence of different phenolic compounds present in the extract. Phenolic compounds are believed to exert their antitumor activities by enhancing the production of free radicals and lipid peroxides, which can in turn abolish proteins and enzymes as well as ATP production in cancer cells, thus finally triggering apoptosis process in cancer cells [37]. Furthermore, Gibellini et al. [38] reported that quercetin has cytotoxic activity *in vitro*. Our current chemical analysis revealed the presence of quercetin in the *C. ignea* extract. Also, we have previously reported on the cytotoxic activities of a novel coumarin isolated form *C. ignea* extract against non-small cell lung cancers (Non-SCLC, H460 and H23), as well as human hepatocellular carcinoma cell line, Huh-7 [13].

In the present study, the decrease in the body weight of B(a)P-treated animals may be due to reduced food intake and/or malabsorption, which led to the wasting of skeletal muscles and adipose tissues [39]. Furthermore, a considerable increase in RLW was observed, which may be due to the accumulation of inflammatory cells and the increased proliferation of cancerous cells in the lung [40]. These findings are consistent with previous studies [20,41]. The increase in body weight and the reduction in RLW of *C. ignea* pre- and post-treatment groups indicated its protective efficacy, which may be attributed to the inhibitory effect of this extract on inflammation, proliferation, and tumor growth. These findings were confirmed by the histopathological observation of lung tissues.

Serum ADA activity, as a tumor marker, is found to be increased in patients with lung cancer [42]. ADA is one of the main enzymes in the purine metabolism; it catalyzes the irreversible deamination of adenosine to inosine [43], which can then be deribosylated and converted to hypoxanthine. Hypoxanthine is an important intermediate in the synthesis of the purine nucleotides *via* the salvage pathways. These nucleotides are required by rapidly proliferating cancer cells for DNA synthesis [44]. The elevated ADA activity observed in B(a)P-treated mice reveals an accelerated salvage pathway, which provides more nucleotides to highly proliferating cancer cells. Pre and post-treatment with *C. ignea* suppressed the elevation of ADA activity in B(a)P-treated mice, which consequently decreased the proliferation rate of cancer cells in both groups, thereby highlighting the antiproliferative effect of *C. ignea* extract.

AHH, also known as cytochrome P4501A1, is a valuable biomarker for the early diagnosis of lung cancer [45]. The activity and expression of AHH are induced through the activation of the aryl hydrocarbon receptor (AhR) by PAHs [46]. Procarcinogen B(a)P is biotransformed by AHH to its ultimate carcinogenic intermediate, (±)-anti-benzo[a]pyrene-7,8-diol-9,10-epoxide (BPDE), which forms mutagenic adducts with DNA, thereby initiating cancer [47]. The B(a)P-treated group had elevated AHH activity, which agreed with the results of Anandakumar et al. [48]. This elevation was reduced by *C. ignea* supplementation in pre- and post-treated animals. The decrease in AHH activity may occur through the inhibition of B(a)P binding to AhR, which results in decreased B(a)P metabolism and inhibits the formation of the carcinogenic reactive intermediate BPDE. The anti-tumorigenic activity of *C. ignea* extract may be due to the presence of quercetin in the extract. This result agreed with Jin et al. [49], who reported that several phytochemicals, including quercetin, modulates AHH activity through the inhibition of B(a)P binding to AhR.

Serum LDH is a prognostic biomarker for many types of cancer as an elevated level of LDH activity was observed in many malignant tumors [50]. LDH has an important role in the regulation of glycolysis, which is the only energy-producing pathway for accelerated tumor growth [51]. Xie et al. [52] determined that LDH was important for the survival and proliferation of cancer cells. In the present study, the observed increase in the serum LDH level in the B(a)P group could be due to an increase in glycolysis [41]. Anandakumar et al. [53] observed a high rate of glycolysis in proliferating malignant cells, which led to an increase in LDH activity. *C. ignea* treatment reduced the activity level of LDH to nearly normal levels, particularly in the case of pre-treatment.

Protein kinase C (PKC) isozymes are a family of 10 structurally related serine/threonine kinases with different biological functions. The PKC family is implicated in signal transduction pathways that are essential for cell proliferation and differentiation [7]. PKCα is a signaling protein that plays a vital role in cancer cell proliferation, apoptosis, and differentiation. It can participate directly or indirectly in the progression of tumors of different cell types [54]. In the present study, the expression of PKCα was elevated in the B(a)P group. In line with our findings, Garg et al. [54] demonstrated that the expression of PKCα was elevated in lung adenocarcinoma compared with nonmalignant lung tissue. *C. ignea* pre- and post-treatment down regulated the expression of PKCα.

The metabolism of B(a)P produces enormous amounts of ROS, which are then involved in the mediation of tissue lipid peroxidation. MDA, a lipid peroxidation product, has been shown to be mutagenic and carcinogenic, as it is involved in the formation of MDA-DNA adducts that undergo genetic mutations responsible for the initiation of carcinogenesis [48,55]. In the present study, MDA levels were significantly increased in the lung tissues of B(a)P-treated mice, which may be due to the excessive amounts of ROS produced in response to B(a)P administration [56]. Pre- and post-supplementation with *C. ignea* reduced MDA levels, which indicated its antioxidant effect against B(a)P-induced lipid peroxidation.

GSH, a tripeptide non-protein thiol, is the major endogenous non-enzymatic antioxidant defense system that acts as a free radical scavenger to combat ROS generation [57]. In this study, the B(a)P-treated group showed lower levels of GSH and TAC. In accordance with the present findings, Kumar et al. [58] reported a depletion in the GSH level in the lung tissues of the B(a)P group, which may be caused by its consumption by lung cells, in conjugation with BPDE, and its involvement as an antioxidant against ROS-induced lipid peroxidation. In contrast, *C. ignea* treatment increased the tissue levels of TAC and GSH, which may be related to its potent antioxidant activity against ROS.

The antioxidant enzymes are scavengers of free radicals, which form a supportive team of cellular antioxidant defenses against oxidative damage induced by ROS [59]. In the current study, the significant decrease in the activity levels of antioxidant enzymes (GSH-Px and CAT) in the lung tissues of B(a)P-treated mice may be attributed to the utilization of these enzymes in the elimination of ROS and the prevention of lipid peroxidation induced during oxidative stress [20]. However, treatment with *C. ignea* extract improved the activity of these antioxidant enzymes, which indicated the free radical scavenging activity of *C. ignea* extract against B(a)P-induced free radicals and hence protects the lung tissues from oxidative damage. The reduction in the activity levels of antioxidant enzymes and the depletion of the non-enzymatic antioxidants in B(a)P-treated group are the main reasons behind B(a)P-induced oxidative stress, which has an important role in the pathophysiology of B(a)P-induced lung cancer [60]. However, *C. ignea* treatment inhibited B(a)P-induced oxidative stress through the elevation of the levels of both enzymatic and non-enzymatic antioxidants in the lung tissues.

It is clear from the results of the radical scavenging and reducing power assays conducted previously (under publication) that *C. ignea* extract has potent antioxidant activity. This antioxidant activity is due to the presence of considerable amounts of flavonoids and phenolic compounds in the *C. ignea* extract. In addition, a novel coumarin isolated from *C. ignea* extract contributed significantly to these effects. Earlier studies demonstrated that the presence of highly reactive hydroxyl groups in the ring of the flavonoid structure was able to neutralize ROS; therefore, the interruption of free radical propagation and the inhibition of oxidative damage induced by lipid peroxidation [61].

Chronic inflammation has an important role in increasing the risk of cancer development [62] through the activation of a number of molecular pathways; one of these pathways is *via* NF-κB, which is activated in the early stages of carcinogenesis [63]. The transcriptional activation of NF-κB, a crucial inflammatory marker, mediates the induction of pro-inflammatory cytokines, chemokines, COX-2, and MMPs [64]. Moreover, NF-κB blocks apoptosis by enhancing the transcription of anti-apoptotic proteins, such as Bcl-2 [65]. Therefore, NF-κB is considered to be a potential link between inflammation and cancer [66] owing to its ability to upregulate the expression of various genes involved in inflammation, immunity, and proliferation [67]. COX-2 is an inducible form of cyclooxygenase that has a vital role in the inflammatory signaling pathways and has been involved in carcinogenic processes [68]. The expression of COX-2 is induced by different stimuli, such as growth factors and cytokines [69]; COX-2 is overexpressed in different types of human cancers and has been shown to induce angiogenesis and increase cellular invasion [70]. A high expression of COX2 was observed in 85% of lung cancers [71]. Our results revealed that the generation of inflammatory responses in the B(a)P group was clearly mediated through the increase of NF-κB and the upregulation of COX-2. These results were consistent with previous studies [20]. *C. ignea* reduced inflammation in the lungs when supplemented as both pre- and post-treatments, partially through the reduction of the elevation in NF-κB and the downregulation of COX-2 level. These findings confirmed that the anti-inflammatory effect of *C. ignea* extract may be due to its polyphenolic content. Polyphenols exerted their anti-inflammatory activity through the modulation of NF-κB activation, the inhibition of the expression of pro-inflammatory enzymes such as nitric oxide synthase and COX-2, and the decreased expression of pro-inflammatory cytokines (TNF-α, IL-1β, and IL-6). Furthermore, the free radical scavenging activity of polyphenols may contribute to their anti-inflammatory effects through a reduction in the formation of ROS, which may initiate the inflammatory process [72].

MMPs are a family of proteolytic enzymes that are able to degrade the extracellular matrix and basement membranes [73]. The elevated activity levels of MMPs may be due to high concentration of ROS; these are the main regulators of MMP activation under prolonged oxidative stress [74]. MMP-2 (gelatinase A) is one of the MMP subgroups that selectively degrade type IV collagen [75] and it plays a key role in tumor invasion, metastasis, and angiogenesis [76]. In the present study, an elevated level of MMP-2 was observed in the B(a)P-treated group, which was in agreement with other studies [77]. Treatment with *C. ignea* decreased the activity levels of MMP-2, and thereby reduced the possibility of tumor progression mediated by MMP-2. Hence, the inhibition of MMP-2 mediated invasion could be an important feature for cancer prevention [77].

MMP-12 is an elastolytic MMP released by the activated neutrophils [78] and is able to degrade elastin fibers in the lung [79]. According to Merchant et al. [80], the overexpression of MMP-12 in lung adenocarcinoma leads to inflammatory cell infiltration and increased epithelial growth. The observed increase in MMP-12 activity in the B(a)P group agreed with the findings of Houghton et al. [81]. According to Moroy et al. [82], the elevated level of MMP-12 was directly related to uncontrolled tumor proliferation, cancer invasion, and metastasis in lung cancer. The present data showed that the elevation of MMP-12 activity was reduced by *C. ignea* treatment.

Angiogenesis is necessary for tumor growth and metastasis [74]. ROS is one of the many stimuli that activate angiogenesis *in vivo* through the release of angiogenic factors by tumor or inflammatory cells into the tumor microenvironment. VEGF is a major growth factor that prompts angiogenesis through the formation of a new vascular network of blood vessels. These blood vessels stimulate endothelial cell proliferation, migration and differentiation [83]. The elevation of VEGF in the B(a)P group was consistent with the findings of Yeo et al. [84]. Previously, Huang et al. [85] reported that VEGF has an important role in tumor progression through the stimulation of angiogenesis. The observed increase in VEGF level in the B(a)P group was attenuated by *C. ignea*, which inhibited tumor growth through its suppressive effect on VEGF in the lungs of groups with both pre- and post-treatment supplementation.

Apoptosis is a programmed cell death process that removes abnormal cells with damaged DNA from the cellular system [86]. The intrinsic apoptotic pathway is regulated by members of the Bcl-2 family. This family of structurally and functionally related proteins contains pro-apoptotic and anti-apoptotic members that interact to prevent or induce apoptosis [87]. BAX, a pro-apoptotic, and Bcl-2, an anti-apoptotic member of the Bcl-2 family form a Bcl-2/BAX heterodimer, which is the main step in the regulation of apoptosis through caspase enzymes [88].The early stages of apoptosis are characterized by the activation of caspase enzymes, which play a vital role in cell death [89]. Caspase-3, as an effector caspase, participates in the execution phase of the apoptotic pathway [90] and is a mediator of DNA fragmentation [91]. One of the distinctive features of the late events of apoptosis is the degradation of DNA to oligonucleosomal fragments, which are found on a DNA-agarose gel as an exclusive ladder of DNA fragments of 180–200 base pairs [92]. In the present study, the oral administration of B(a)P to mice inhibited apoptosis, and shifted the cell balance toward cellular proliferation through the decreased expression of BAX and caspase-3, combined with an increase in Bcl-2 expression that was consistent with the reports of Nair et al. [93]. In addition, our results agreed with Hassan et al. [94], who reported that the interruption of apoptosis could be an early occurrence in carcinogenesis. Our data showed that treatment with *C. ignea* extract activated the intrinsic pathway of apoptosis, which is characterized by the upregulation of the expression of BAX and caspase-3 and the downregulation of the expression of Bcl-2, leading to a low Bcl-2/BAX ratio. Nair et al. [93] reported that a low Bcl-2/BAX ratio promotes caspase-mediated apoptosis. In addition, an increase in apoptotic DNA fragments was observed on an agarose gel in the *C. ignea* pre- and post-treatment groups. The apoptotic effect of the extract may be due to the presence of polyphenols in the extract. Polyphenols activate caspases, upregulate the expression of pro-apoptotic proteins, and downregulate the expression of anti-apoptotic proteins [95]. Thus, the induction of apoptosis may be one of the molecular events responsible for the chemopreventive effect of *C. ignea* extract.

## 5 Conclusion

The results of the present study support the promising role of the aqueous ethanolic extract of the aerial parts of *C. ignea* as a chemopreventive agent against B(a)P-induced lung tumorigenesis in mouse model, and confirmed that pre-treatment was more efficacious than post-treatment. The chemopreventive potential of *C. ignea* extract may be due to its ability to combat B(a)P-induced oxidative stress in the lung tissues through the amelioration of the antioxidant defense system. This *in vitro* study clearly revealed that the *C. ignea* extract has cytotoxic activity against human lung cancer cell line (A549). Collectively, the results of the *in vitro* and *in vivo* studies have demonstrated the effectiveness of *C. ignea* extract in the management of lung tumorigenesis. However, further detailed investigations are needed in the future to elucidate the possible molecular mechanisms underlying the observed antitumor effect.

## Author contributions

SKH and AMM proposed the research concept and designed the experimental model. SKH, AMM, NME, and AHA performed the animal studies, all *in vivo* animal experiments, and analyzed and interpreted the data. WKBK performed the molecular studies. EAE conducted *in vitro* studies, analyzed the data, and corresponded the manuscript. NA performed the histological analysis. SKH and AMM wrote the manuscript. EAE, NME, and AHA contributed to the revision of the manuscript. ESM and ANH performed the collection and extraction of the plant. MWL, ANH, MAN, and ESM isolated, purified, and identified the phenolic compounds of the plant extract. All authors reviewed and approved the final manuscript.

## Declaration of Competing Interest

The authors declare that they have no conflict of interest.
